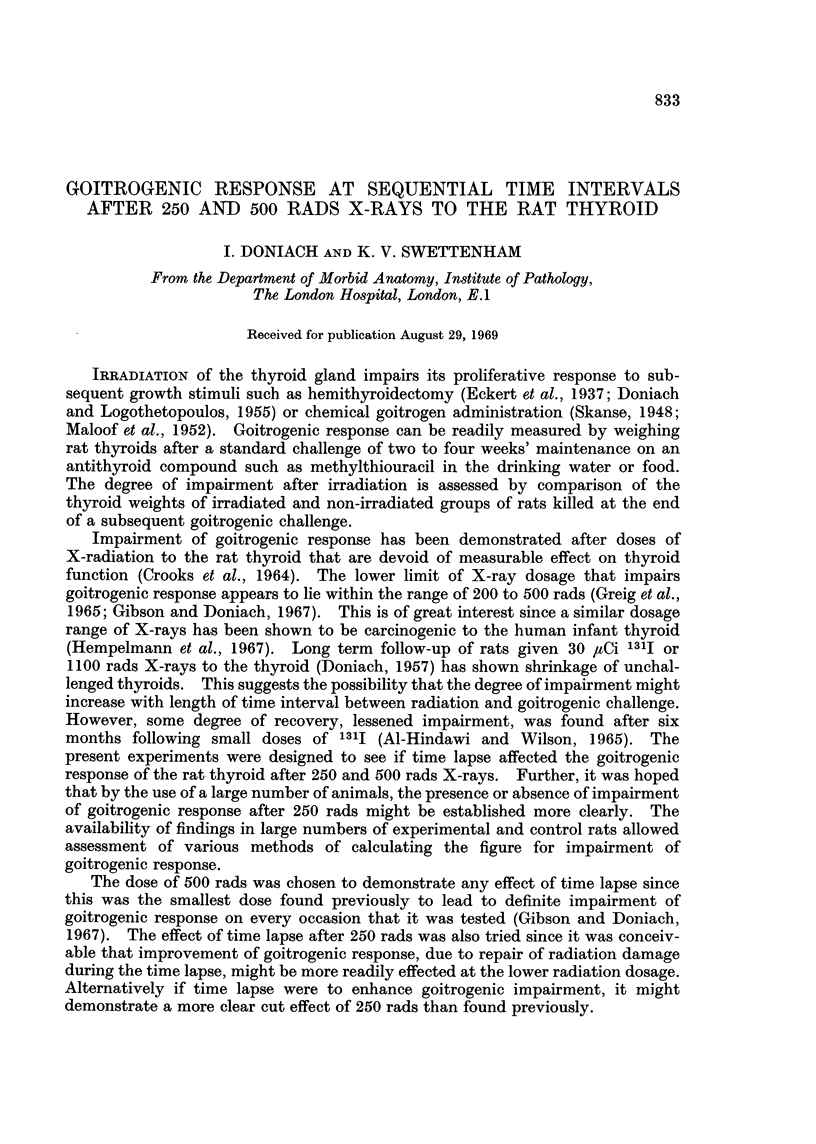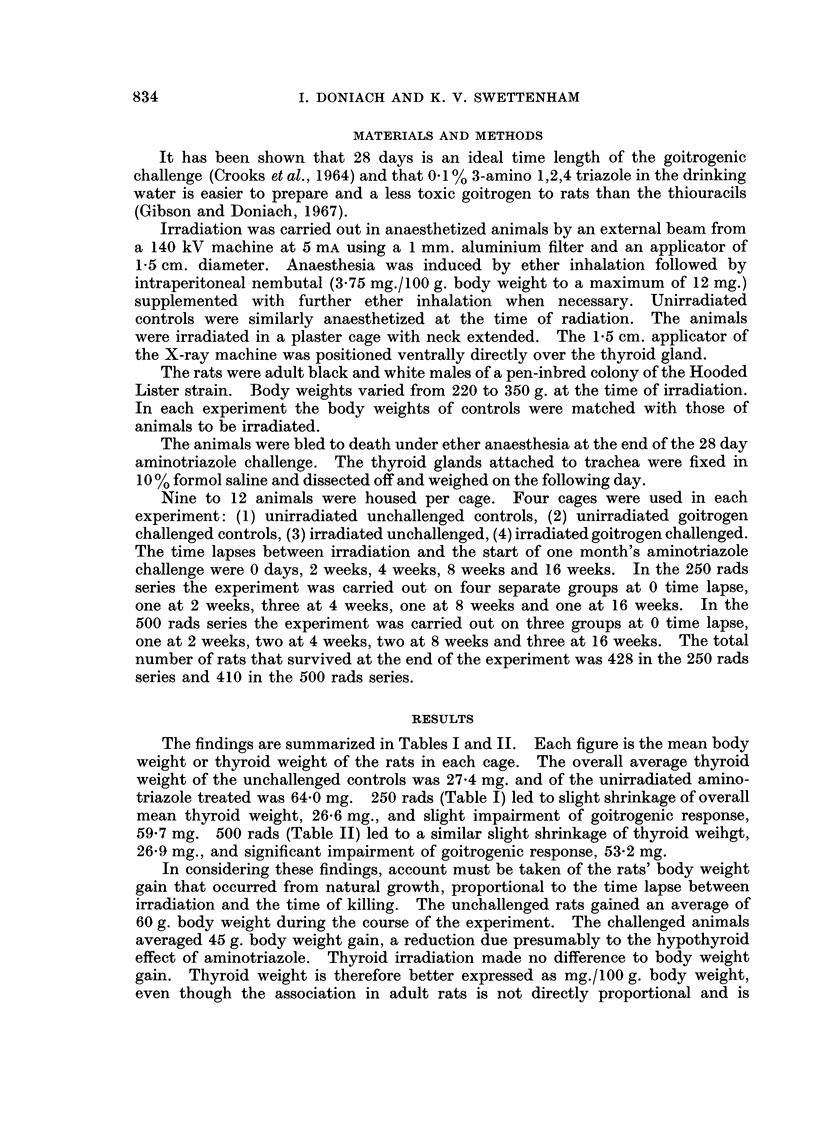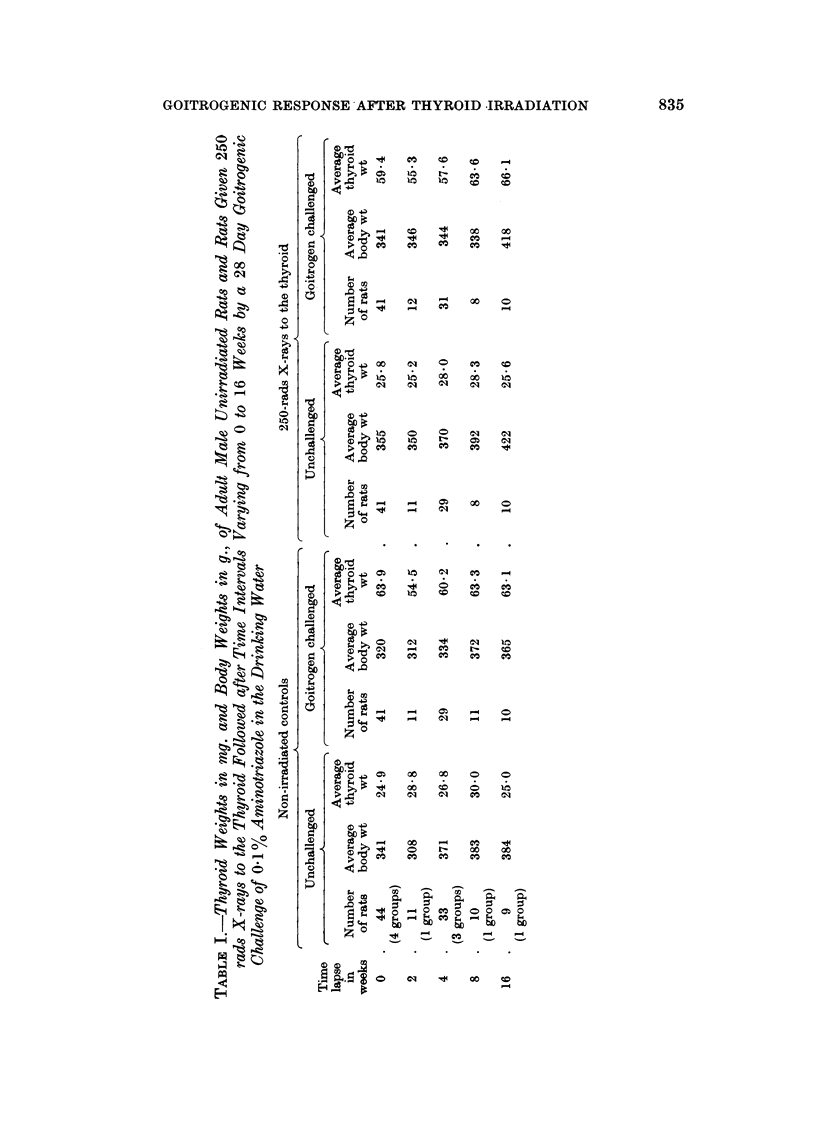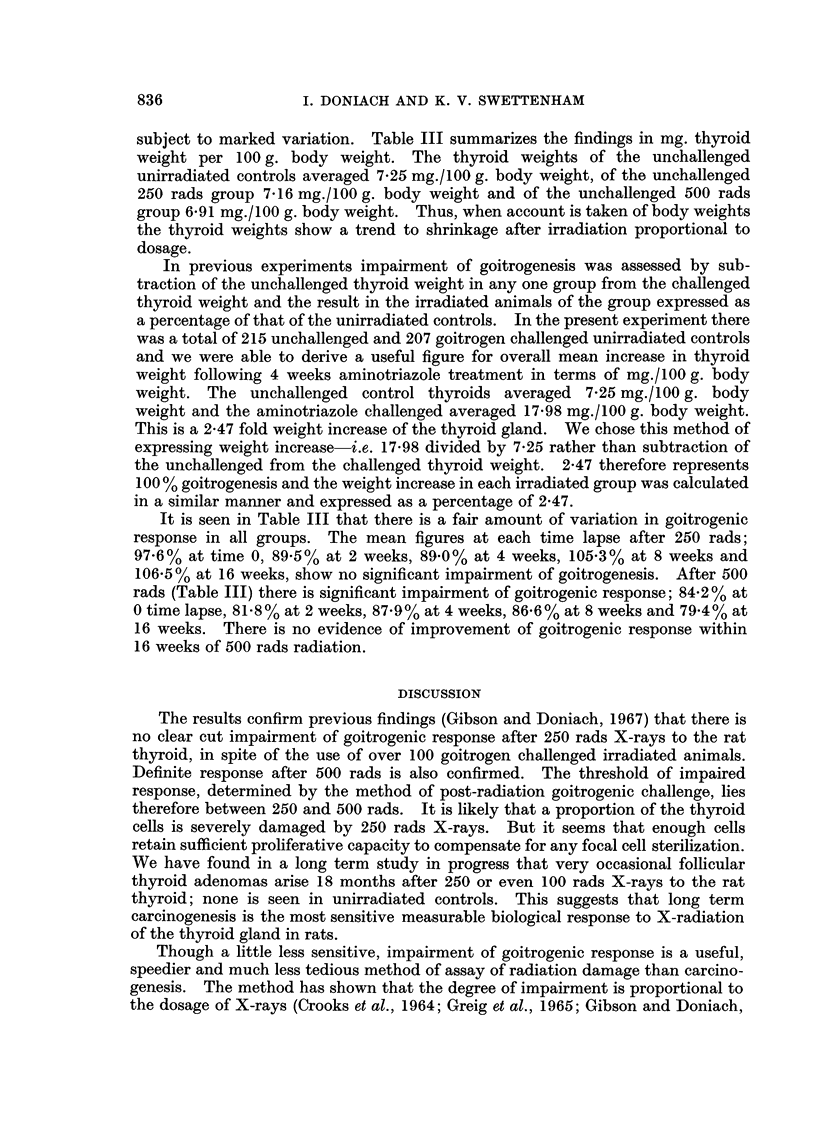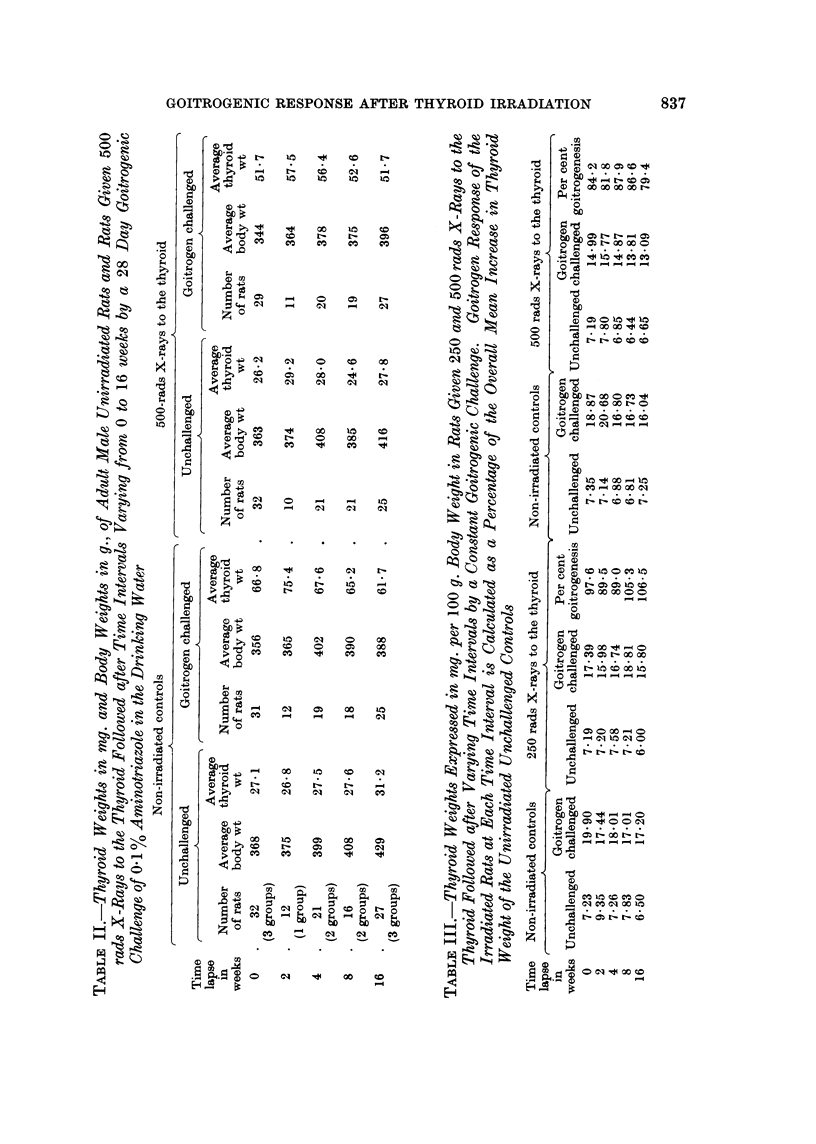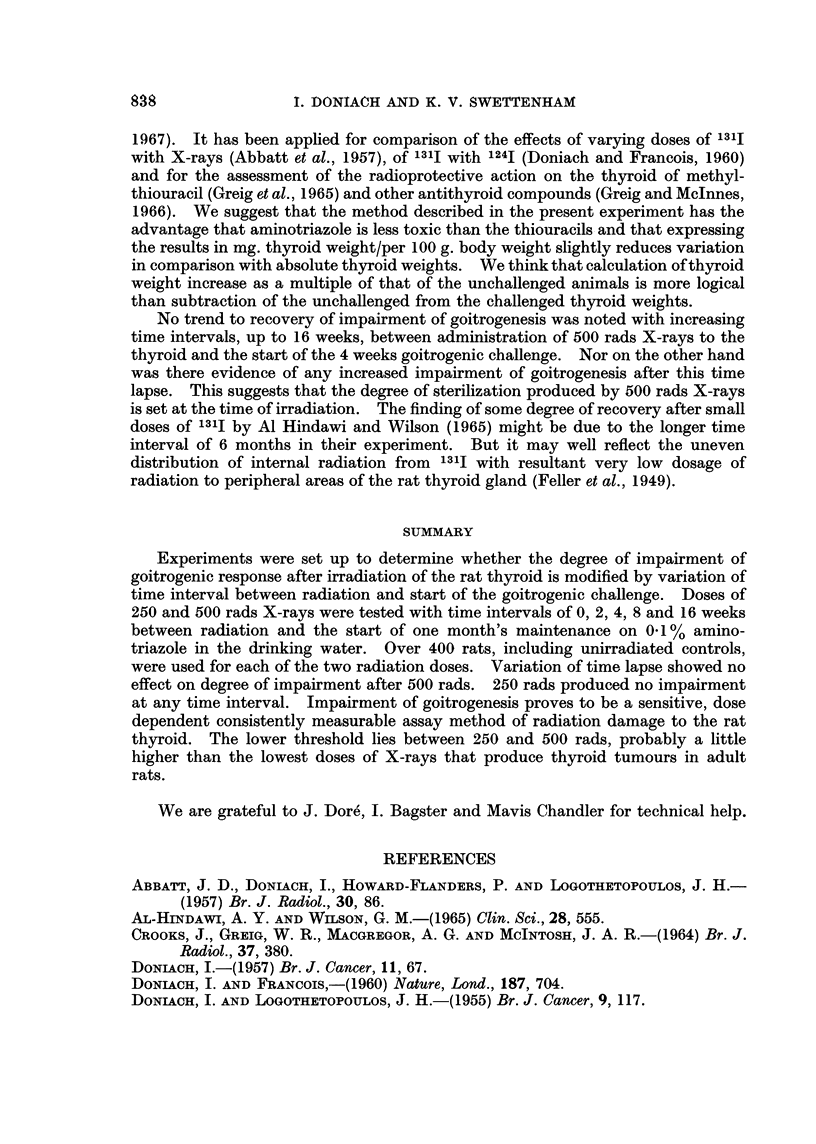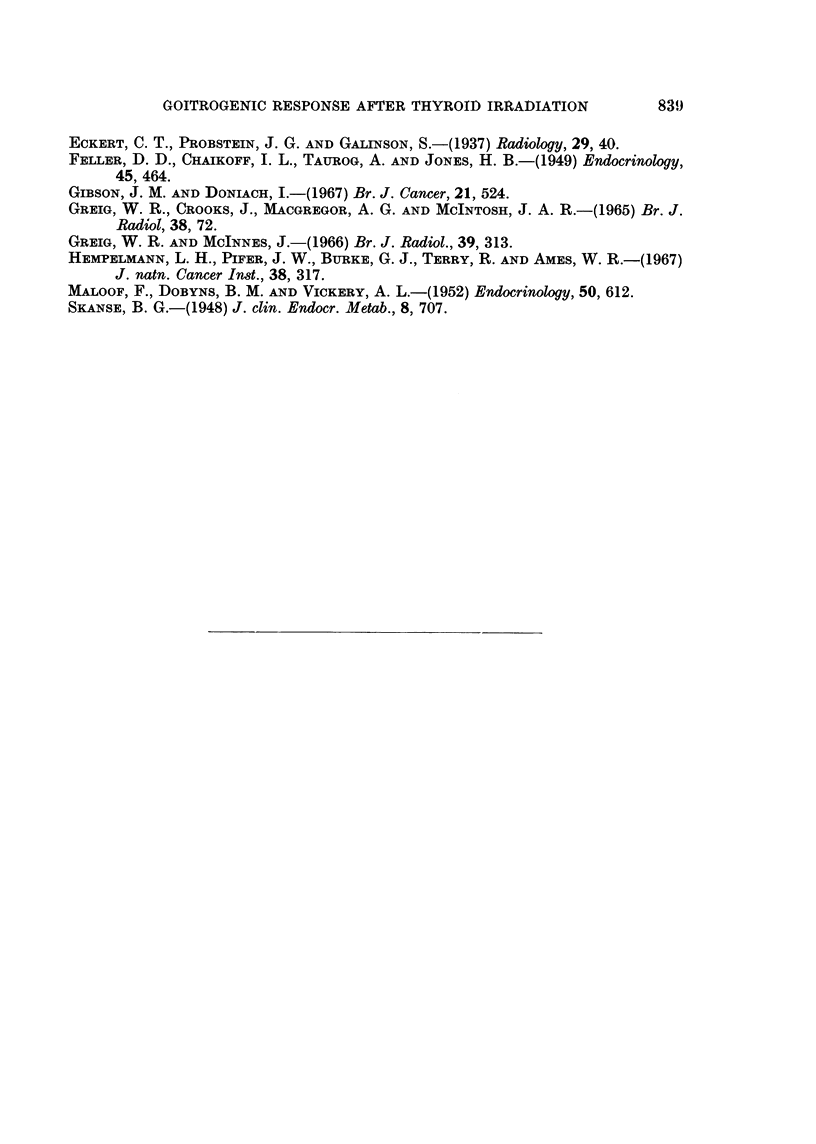# Goitrogenic response at sequential time intervals after 250 and 500 rads x-rays to the rat thyroid.

**DOI:** 10.1038/bjc.1969.103

**Published:** 1969-12

**Authors:** I. Doniach, K. V. Sweetenham


### GOITROGENIC RESPONSE AT SEQUENTIAL TIME INTERVALS AFTER 250 AND 500 RADS X-RAYS TO THE RAT THYROID

I. DONIACH AND K. V. SWETTENHAM

*From the Department of Morbid Anatomy, Institute of Pathology, The London Hospital, London, E.1*

Received for publication August 29, 1969

IRRADIATION of the thyroid gland impairs its proliferative response to subsequent growth stimuli such as hemithyroidectomy (Eckert *et al.*, 1937; Doniach and Logothetopoulos, 1955) or chemical goitrogen administration (Skanse, 1948; Maloof *et al.*, 1952). Goitrogenic response can be readily measured by weighing rat thyroids after a standard challenge of two to four weeks' maintenance on an antithyroid compound such as methylthiouracil in the drinking water or food. The degree of impairment after irradiation is assessed by comparison of the thyroid weights of irradiated and non-irradiated groups of rats killed at the end of a subsequent goitrogenic challenge.

Impairment of goitrogenic response has been demonstrated after doses of X-radiation to the rat thyroid that are devoid of measurable effect on thyroid function (Crooks *et al.*, 1964). The lower limit of X-ray dosage that impairs goitrogenic response appears to lie within the range of 200 to 500 rads (Greig *et al.*, 1965; Gibson and Doniach, 1967). This is of great interest since a similar dosage range of X-rays has been shown to be carcinogenic to the human infant thyroid (Hempelmann *et al.*, 1967). Long term follow-up of rats given 30 $\mu$Ci $^{131}$I or 1100 rads X-rays to the thyroid (Doniach, 1957) has shown shrinkage of unchallenged thyroids. This suggests the possibility that the degree of impairment might increase with length of time interval between radiation and goitrogenic challenge. However, some degree of recovery, lessened impairment, was found after six months following small doses of $^{131}$I (Al-Hindawi and Wilson, 1965). The present experiments were designed to see if time lapse affected the goitrogenic response of the rat thyroid after 250 and 500 rads X-rays. Further, it was hoped that by the use of a large number of animals, the presence or absence of impairment of goitrogenic response after 250 rads might be established more clearly. The availability of findings in large numbers of experimental and control rats allowed assessment of various methods of calculating the figure for impairment of goitrogenic response.

The dose of 500 rads was chosen to demonstrate any effect of time lapse since this was the smallest dose found previously to lead to definite impairment of goitrogenic response on every occasion that it was tested (Gibson and Doniach, 1967). The effect of time lapse after 250 rads was also tried since it was conceivable that improvement of goitrogenic response, due to repair of radiation damage during the time lapse, might be more readily effected at the lower radiation dosage. Alternatively if time lapse were to enhance goitrogenic impairment, it might demonstrate a more clear cut effect of 250 rads than found previously.

### MATERIALS AND METHODS

It has been shown that 28 days is an ideal time length of the goitrogenic challenge (Crooks *et al.*, 1964) and that 0·1% 3-amino 1,2,4 triazole in the drinking water is easier to prepare and a less toxic goitrogen to rats than the thiouracils (Gibson and Doniach, 1967).

Irradiation was carried out in anaesthetized animals by an external beam from a 140 kV machine at 5 mA using a 1 mm. aluminium filter and an applicator of 1·5 cm. diameter. Anaesthesia was induced by ether inhalation followed by intraperitoneal nembutal (3·75 mg./100 g. body weight to a maximum of 12 mg.) supplemented with further ether inhalation when necessary. Unirradiated controls were similarly anaesthetized at the time of radiation. The animals were irradiated in a plaster cage with neck extended. The 1·5 cm. applicator of the X-ray machine was positioned ventrally directly over the thyroid gland.

The rats were adult black and white males of a pen-inbred colony of the Hooded Lister strain. Body weights varied from 220 to 350 g. at the time of irradiation. In each experiment the body weights of controls were matched with those of animals to be irradiated.

The animals were bled to death under ether anaesthesia at the end of the 28 day aminotriazole challenge. The thyroid glands attached to trachea were fixed in 10% formol saline and dissected off and weighed on the following day.

Nine to 12 animals were housed per cage. Four cages were used in each experiment: (1) unirradiated unchallenged controls, (2) unirradiated goitrogen challenged controls, (3) irradiated unchallenged, (4) irradiated goitrogen challenged. The time lapses between irradiation and the start of one month's aminotriazole challenge were 0 days, 2 weeks, 4 weeks, 8 weeks and 16 weeks. In the 250 rads series the experiment was carried out on four separate groups at 0 time lapse, one at 2 weeks, three at 4 weeks, one at 8 weeks and one at 16 weeks. In the 500 rads series the experiment was carried out on three groups at 0 time lapse, one at 2 weeks, two at 4 weeks, two at 8 weeks and three at 16 weeks. The total number of rats that survived at the end of the experiment was 428 in the 250 rads series and 410 in the 500 rads series.

### RESULTS

The findings are summarized in Tables I and II. Each figure is the mean body weight or thyroid weight of the rats in each cage. The overall average thyroid weight of the unchallenged controls was 27·4 mg. and of the unirradiated aminotriazole treated was 64·0 mg. 250 rads (Table I) led to slight shrinkage of overall mean thyroid weight, 26·6 mg., and slight impairment of goitrogenic response, 59·7 mg. 500 rads (Table II) led to a similar slight shrinkage of thyroid weihgt, 26·9 mg., and significant impairment of goitrogenic response, 53·2 mg.

In considering these findings, account must be taken of the rats' body weight gain that occurred from natural growth, proportional to the time lapse between irradiation and the time of killing. The unchallenged rats gained an average of 60 g. body weight during the course of the experiment. The challenged animals averaged 45 g. body weight gain, a reduction due presumably to the hypothyroid effect of aminotriazole. Thyroid irradiation made no difference to body weight gain. Thyroid weight is therefore better expressed as mg./100 g. body weight, even though the association in adult rats is not directly proportional and is subject to marked variation. Table III summarizes the findings in mg. thyroid weight per 100 g. body weight. The thyroid weights of the unchallenged unirradiated controls averaged 7·25 mg./100 g. body weight, of the unchallenged 250 rads group 7·16 mg./100 g. body weight and of the unchallenged 500 rads group 6·91 mg./100 g. body weight. Thus, when account is taken of body weights the thyroid weights show a trend to shrinkage after irradiation proportional to dosage.

In previous experiments impairment of goitrogenesis was assessed by subtraction of the unchallenged thyroid weight in any one group from the challenged thyroid weight and the result in the irradiated animals of the group expressed as a percentage of that of the unirradiated controls. In the present experiment there was a total of 215 unchallenged and 207 goitrogen challenged unirradiated controls and we were able to derive a useful figure for overall mean increase in thyroid weight following 4 weeks aminotriazole treatment in terms of mg./100 g. body weight. The unchallenged control thyroids averaged 7·25 mg./100 g. body weight and the aminotriazole challenged averaged 17·98 mg./100 g. body weight. This is a 2·47 fold weight increase of the thyroid gland. We chose this method of expressing weight increase—*i.e.* 17·98 divided by 7·25 rather than subtraction of the unchallenged from the challenged thyroid weight. 2·47 therefore represents 100% goitrogenesis and the weight increase in each irradiated group was calculated in a similar manner and expressed as a percentage of 2·47.

It is seen in Table III that there is a fair amount of variation in goitrogenic response in all groups. The mean figures at each time lapse after 250 rads; 97·6% at time 0, 89·5% at 2 weeks, 89·0% at 4 weeks, 105·3% at 8 weeks and 106·5% at 16 weeks, show no significant impairment of goitrogenesis. After 500 rads (Table III) there is significant impairment of goitrogenic response; 84·2% at 0 time lapse, 81·8% at 2 weeks, 87·9% at 4 weeks, 86·6% at 8 weeks and 79·4% at 16 weeks. There is no evidence of improvement of goitrogenic response within 16 weeks of 500 rads radiation.

### DISCUSSION

The results confirm previous findings (Gibson and Doniach, 1967) that there is no clear cut impairment of goitrogenic response after 250 rads X-rays to the rat thyroid, in spite of the use of over 100 goitrogen challenged irradiated animals. Definite response after 500 rads is also confirmed. The threshold of impaired response, determined by the method of post-radiation goitrogenic challenge, lies therefore between 250 and 500 rads. It is likely that a proportion of the thyroid cells is severely damaged by 250 rads X-rays. But it seems that enough cells retain sufficient proliferative capacity to compensate for any focal cell sterilization. We have found in a long term study in progress that very occasional follicular thyroid adenomas arise 18 months after 250 or even 100 rads X-rays to the rat thyroid; none is seen in unirradiated controls. This suggests that long term carcinogenesis is the most sensitive measurable biological response to X-radiation of the thyroid gland in rats.

Though a little less sensitive, impairment of goitrogenic response is a useful, speedier and much less tedious method of assay of radiation damage than carcinogenesis. The method has shown that the degree of impairment is proportional to the dosage of X-rays (Crooks *et al.*, 1964; Greig *et al.*, 1965; Gibson and Doniach,

1967). It has been applied for comparison of the effects of varying doses of $^{131}I$ with X-rays (Abbatt *et al.*, 1957), of $^{131}I$ with $^{124}I$ (Doniach and Francois, 1960) and for the assessment of the radioprotective action on the thyroid of methylthiouracil (Greig *et al.*, 1965) and other antithyroid compounds (Greig and McInnes, 1966). We suggest that the method described in the present experiment has the advantage that aminotriazole is less toxic than the thiouracils and that expressing the results in mg. thyroid weight/per 100 g. body weight slightly reduces variation in comparison with absolute thyroid weights. We think that calculation of thyroid weight increase as a multiple of that of the unchallenged animals is more logical than subtraction of the unchallenged from the challenged thyroid weights.

No trend to recovery of impairment of goitrogenesis was noted with increasing time intervals, up to 16 weeks, between administration of 500 rads X-rays to the thyroid and the start of the 4 weeks goitrogenic challenge. Nor on the other hand was there evidence of any increased impairment of goitrogenesis after this time lapse. This suggests that the degree of sterilization produced by 500 rads X-rays is set at the time of irradiation. The finding of some degree of recovery after small doses of $^{131}I$ by Al Hindawi and Wilson (1965) might be due to the longer time interval of 6 months in their experiment. But it may well reflect the uneven distribution of internal radiation from $^{131}I$ with resultant very low dosage of radiation to peripheral areas of the rat thyroid gland (Feller *et al.*, 1949).

### SUMMARY

Experiments were set up to determine whether the degree of impairment of goitrogenic response after irradiation of the rat thyroid is modified by variation of time interval between radiation and start of the goitrogenic challenge. Doses of 250 and 500 rads X-rays were tested with time intervals of 0, 2, 4, 8 and 16 weeks between radiation and the start of one month's maintenance on 0·1% aminotriazole in the drinking water. Over 400 rats, including unirradiated controls, were used for each of the two radiation doses. Variation of time lapse showed no effect on degree of impairment after 500 rads. 250 rads produced no impairment at any time interval. Impairment of goitrogenesis proves to be a sensitive, dose dependent consistently measurable assay method of radiation damage to the rat thyroid. The lower threshold lies between 250 and 500 rads, probably a little higher than the lowest doses of X-rays that produce thyroid tumours in adult rats.

We are grateful to J. Doré, I. Bagster and Mavis Chandler for technical help.

### REFERENCES

ABBATT, J. D., DONIACH, I., HOWARD-FLANDERS, P. AND LOGOTHETOPOULOS, J. H.—(1957) *Br. J. Radiol.*, **30**, 86.

AL-HINDAWI, A. Y. AND WILSON, G. M.—(1965) *Clin. Sci.*, **28**, 555.

CROOKS, J., GREIG, W. R., MACGREGOR, A. G. AND MCINTOSH, J. A. R.—(1964) *Br. J. Radiol.*, **37**, 380.

DONIACH, I.—(1957) *Br. J. Cancer*, **11**, 67.

DONIACH, I. AND FRANCOIS,—(1960) *Nature, Lond.*, **187**, 704.

DONIACH, I. AND LOGOTHETOPOULOS, J. H.—(1955) *Br. J. Cancer*, **9**, 117.

ECKERT, C. T., PROBSTEIN, J. G. AND GALINSON, S.—(1937) *Radiology*, **29**, 40.
FELLER, D. D., CHAIKOFF, I. L., TAUROG, A. AND JONES, H. B.—(1949) *Endocrinology*, **45**, 464.
GIBSON, J. M. AND DONIACH, I.—(1967) *Br. J. Cancer*, **21**, 524.
GREIG, W. R., CROOKS, J., MACGREGOR, A. G. AND MCINTOSH, J. A. R.—(1965) *Br. J. Radiol*, **38**, 72.
GREIG, W. R. AND MCINNES, J.—(1966) *Br. J. Radiol.*, **39**, 313.
HEMPELMANN, L. H., PIFER, J. W., BURKE, G. J., TERRY, R. AND AMES, W. R.—(1967) *J. natn. Cancer Inst.*, **38**, 317.
MALOOF, F., DOBYNS, B. M. AND VICKERY, A. L.—(1952) *Endocrinology*, **50**, 612.
SKANSE, B. G.—(1948) *J. clin. Endocr. Metab.*, **8**, 707.